# Effect of local injection of Zolena, zoledronic acid made in Iran, on orthodontic tooth movement and root and bone resorption in rats

**DOI:** 10.15171/joddd.2017.045

**Published:** 2017-12-13

**Authors:** Massoud Seifi, Sohrab Asefi, Ghazal Hatamifard, Ali Lotfi

**Affiliations:** ^1^Department of Restorative Dentistry, Hamadan University of Medical Sciences, Hamadan, Iran; ^2^Department of Orthodontics, International School of Dentistry, Tehran University of Medical Sciences, Tehran, Iran; ^3^Faculty of Science and Technology, Life Sciences Department, Applied Biotechnology Research group, University of Westminster, London, United Kingdom; ^4^Department of Oral and Maxillofacial Pathology, School of Dentistry, Shahid Beheshti University of Medical Sciences, Tehran, Iran

**Keywords:** Bone resorption, rat, root resorption, tooth movement, zolendronic acid

## Abstract

***Background.*** Anchorage control is an essential part of orthodontic treatment planning, especially in adult patients who demand a more convenient treatment. Zoledronic acid (ZA) is an effective choice to address this problem. It is the most potent member of the bisphosphonates family that has an inhibitory effect on bone resorption by suppressing osteoclast function. Therefore, ZA might be a good option for orthodontic anchorage control. The current study evaluated the effect of local administration of Zolena (ZA made in Iran) on orthodontic tooth movement (OTM) and root and bone resorption.

***Methods.*** The experimental group consisted of 30 rats in 3 subgroups (n=10). Anesthesia was induced, and one closed NiTi coil spring was installed between the first molar and central incisor unilaterally, except for the negative control group. The positive control group received vestibular injection of 0.01 mL of saline next to the maxillary first molar, and 0.01 mL of the solution was injected at the same site in the ZA group. After 21 days, the rats were sacrificed and the distance between the first and second molars was measured with a leaf gauge. Histological analysis was conducted by a blind pathologist for the number of Howship’s lacunae, blood vessels, osteoclast-like cells and root resorption lacunae. Data were analyzed with ANOVA, Tukey test and t-test.

***Results.*** There were no significant differences in OTM between the force-applied groups. ZA significantly inhibited bone/root resorption and angiogenesis compared to the positive control group.

***Conclusion.*** Zolena did not decrease OTM but significantly inhibited bone and root resorption. Zolena might be less potent than its foreign counterparts.

## Introduction

Orthodontic tooth movement (OTM) depends on periodontal cells and the interactions between osteoblasts and osteoclasts. Bone remodeling is the basis of OTM. As osteoclasts resorb the old bone, new bone is formed by osteoblasts. This process is regulated by variousfactors.^1-3^

Control of anchorage is an essential part of orthodontic treatment planning,^4^ and involves using extraoral or intraoral appliances to control unfavorable tooth movement. Many appliances have been designed for better anchorage control such as social and peer pressure for extraoral appliances or moderate anchorage preparation for intraoral appliances such as the Nance acrylic button; however, none of them is ideal for this purpose. Accordingly, skeletal anchorage devices have been introduced, which provide absolute anchorage, yet an invasive procedure with high possibility of loosening.^5^ Inthe recent decades, there has been a significant increase in the number of adult orthodontic patients. Hence, less invasive procedures for anchorage control gains importance. Drug prescription, either locally or systemically, is one suggested method for this purpose.^6,7^

Bisphosphonates are the potent inhibitors of bone resorption. They are often prescribed for osteoporosis, resorptive metabolic bone disease or malignancy. The main mechanism of action of bisphosphonates is inhibition of osteoclast function.^8^ They inhibit osteoclast activating factors such as receptor activator of nuclear factor kappa-B ligand (RANKL), which has a major role in bone remodeling.^9^ Therefore, they are likely to slow or suppress orthodontic tooth movement (OTM).

In previous studies,^10-15^ the effect of less potent bisphosphonates was evaluated on OTM and their retentive or anti-relapse effects were confirmed. Among bisphosphonates, zoledronic acid (ZA) has the highest potency being 10-100 times more effective than others.^8^ ZA is a commercially available product which can be supplied from Argentina (Eriophos, Eriochem SA, Argentina) and Switzerland (Zometa and Aclasta, Novartis, Switzerland).According to high-performance liquid chromatography analysis of Zolena in a pilot study, the retention time of Zolena was observed to be about 3 minutes less than that of ZA, which can be found in the literature; this indicates that Zolena can be slightly different in molecular weight. The aim of this study was to evaluate the potential effect of ZA produced in Iran (Zolena) on OTM and root and bone resorption as the first study on this product.

## Methods

This animal study was performed on30normal male Wistar rats (SCL, Shizuoka, Japan). The sample size (n=10 per group) was calculated assuming 0.6 mm significant difference (delta value) in tooth movement between the two independent groups with a conservative estimate of 0.45mm for the standard deviation according to a previous study,^16^ with 80% power and an alpha error rate of 5%. The mean weight of rats was 270± 30 g and their mean age was 8 weeks. The study was approved by the Ethical Committee Board and the experimental procedures were performed in accord with the ARRIVE guideline (Animal Research: Reporting of In vivo Experiments, Available at: www.nc3rs.org.uk/ARRIVE.17). The rats were transferred to the animal room for 2 weeks before the study, in order to acclimatize with their new environment. All the rats received the same nutritional diet and light. Then, they were randomly allocated to three groups of 10 by using a table of random numbers. Each group was colored differently and kept in separate cages.

**Figure-1:**
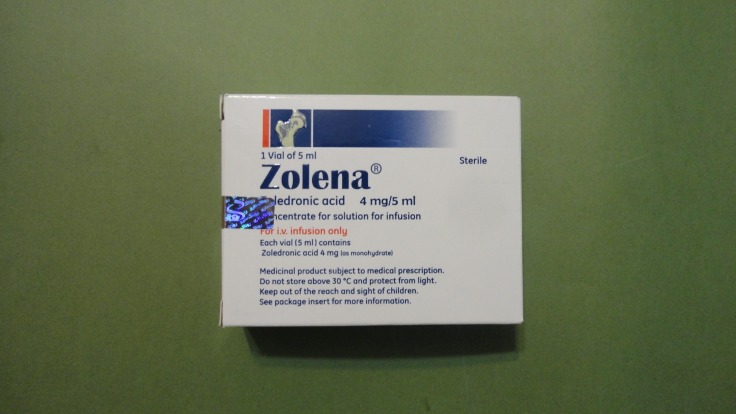
Zolena, 4mg/5ml, Ronak Daroo, Iran.

The three groups consisted of: (1) negative control group, (NC): rats that did not receive any orthodontic appliance and were only anesthetized, (2) positive control group (PC): rats that received orthodontic appliances for their maxillary arch. These rats received 0.01 cc of 0.9% sodium chloride injectable solution (Therapeutic and Injectable Products Company, Tehran, Iran) injected into the vestibule of the mesial root of their maxillary first molars. 3) ZA group (Z): 0.01 cc (approximately 0.02 mg) ZA (Zolena, 4mg/5mL, Ronak Daroo, Iran) was diluted by 0.9%sodium chloride injectable solution (Figure 1) and injected into the vestibule next to the mesial root of the maxillary first molar by a blind operator.

**Figure 2:**
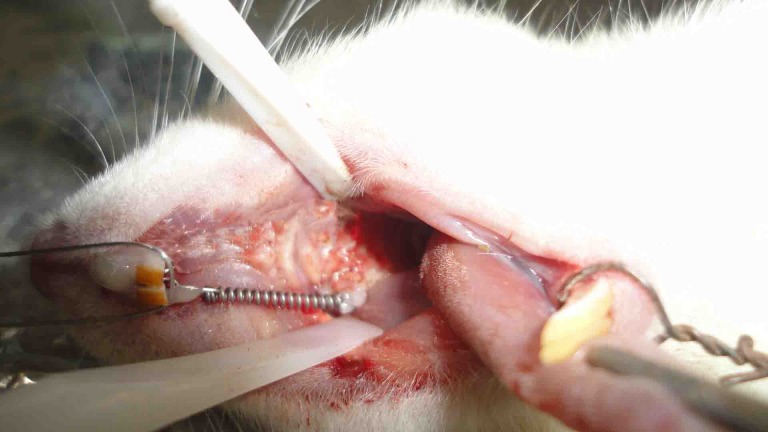
Intraoral view of orthodontic appliance which place between first molar and central incisors.

Rats were weighed by a digital scale (Shimadzu, Kyoto, Japan, 61189). They were anesthetized intraperitoneally using an insulin syringe. The anesthetic solution contained 20 mg/kg of 10% ketamine hydrochloride (Alfasan, Woerden, Holland) and 2 mg/kg of 2% xylazine (Alfasan, Woerden, Holland). Based on the animal care protocol, vital signs were monitored and the room temperature was kept under control. In order to prevent pulmonary edema, rats were rotated from side to side every few minutes. Light orthodontic force was continuously applied by nickel-titanium (Ni-Ti) closed coil springs (American Orthodontics NiTi closed coil, 010x030 inch, 9mm/Eyelet). Springs were fixed between the maxillary first molar and central incisor by 0.01-inch stainless steel ligature wire (3M Unitek, Monrovia, CA, USA). The ligature embraced the first molar across the interdental space and twisted at the mesiobuccal line angle. On the other side of the coil, mechanical retention was obtained by creating a retentive groove on the distolabial line angle of the central incisor using a diamond bur (836, ISO 109/Eur; Tizkavan, Iran) and sterile disposable high speed handpiece (Mehras, T.S.N.P.T Co. Tehran, Iran). Both ligature ties were fixed by light-cure flowable composite resin (DenFil Flow, Vericom Co., Korea) (Figure2). Tipping was the desired orthodontic movement in this study. Injection was done by an insulin syringe into the buccal vestibular mucosa next to the mesial root of the first molar as mentioned earlier, which was desired to move by orthodontic force in mesial direction. The diet was changed to soft diet to prevent appliance dislodgement. The treatment course was considered to be21 days. Then, the rats were weighed and subsequently sacrificed by saturated chloroform inhalation. Rats were decapitated and OTM was measured by a blind operator three times in each group. This was done by measuring the distance between the first and second molars using a leaf gauge (Precision stainless steel feeler gauge, FENGHGO) with 0.05 mm accuracy. The mean of the three measurements was reported as the final value.

The maxilla was removed for histological evaluation and specimens were fixed with10% formalin during 10 days, and 10% formic acid was used for decalcification for 15 days. Then, they were processed with alcohol and ascending concentrations of methyl salicylate solution. Finally, histological specimens were embedded in paraffin blocks and cut into parasagittal sections with a microtome (LEICA, Wetzlar, Germany). The specimens had4-6 µm thickness and they were stained with hematoxylin and eosin. Histological analysis was done by an experienced pathologist who was blinded to the study design and the experimented groups. The number of Howship’s lacunae, blood vessels, osteoblast-like cells and root resorptive lacunae (number and area) were the histological factors assessed. Histological specimens were analyzed under a light microscope (Eclipse E400, Nikon, Japan) and photographed using a camera (E8400, Nikon, Japan) at ×10 and ×40 magnifications in order to perform histomorphometric analysis using NIS-Element software (Nikon, Tokyo, Japan). This was repeated three times. Kolmogorov-Smirnov test showed that the data had normal distribution. Collected data for each specimen were averaged and analyzed using SPSS software (version 21). One-way ANOVA, Tukey test and t-test were used for data analysis.

## Results

**Figure 3:**
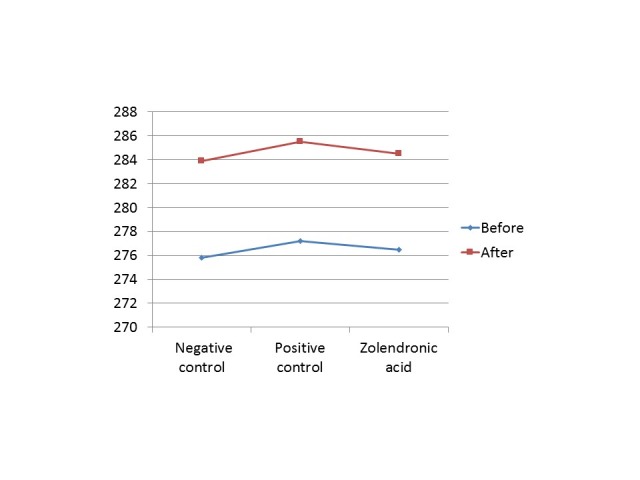
Mean weight of each experimental group before and after ofthe experiment. Experimentdid not change normal development of rats during study.

Figure 3 shows the mean weight of all groups before and after the experiment. The results showed that the experiment and drug administration did not affect normal development of rats.

### Orthodontic tooth movement

Table 1 shows the mean tooth movement in each group. The NC group did not receive any orthodontic appliance; therefore, it was logical to find a significant difference in OTM between this group and others. But, there was no significant difference between PC and Z groups in OTM (P=0.15). The positive control group had the maximum (0.27 mm) and the negative control group showed the minimum (0.01 mm) amount of OTM. ZA did not significantly inhibit tooth movement compared to the PC group.

**Table 1:** The tooth movement measurements (mm)in each group by leaf gauge (0.05 mm accuracy).

**Group**	**Mean**	**Standard Dev.**
**Negative control**	0.015	0.0
**Positive control**	0.27	0.11
**Zolendronic acid**	0.24	0.09

### Histological analysis

Table 2 shows the histological comparison between the groups.

**Table 2:** Histologic analysis of each experiment group.

** Group** **Variables**	**Negative Control**	**Positive Control**	**Zolendronic acid**
**Number of Howship lacunae**	0 ±0.009	4 ±0.040	1 ±0.074
**Number of Blood Vessels**	4 ±0.063	8 ±0.000	5 ±0.080
**Number of Osteoclasts**	0 ±0.005	6 ±0.079	3 ±0.066
**Number of Root Resorptive lacunae**	0 ±0.002	4 ±0.055	1 ± 0.046
**Area of Root Resorptive lacunae**	0.003 ×10^-3^ ±0.000	0.270 ×10^-3^ ±0.013	0.132 ×10^-3^ ±0.005

### The Howship’s lacunae

At the end of orthodontic period, the negative control group did not show any Howship’s lacunae in the test side; however, the PC group showed moderate lacunae formation in presence of active orthodontic force. The ZA group exhibited mild grade of lacunae formation. Therefore, a significant difference was found between groups Z and PC (P=0.02). ZA significantly decreased the number of Howship’s lacunae compared to the PC group.

### Blood vessels

A moderate increase in number of blood vessels was noted in the PC group while the blood vessel distribution was normal in NC and Z groups. A significant difference was found between PC and Z groups in number of blood vessels, which indicated that ZA significantly inhibited angiogenesis (P=0.05).

### Osteoclast-like cells

Group NC showed almost no osteoclastic cell but the PC group exhibited a moderate increase in number of osteoclast-like cells. ZA specimens had normal osteoclast distribution. A significant difference was found between groups Z and PC in this regard (P=0.04).

### Root resorption

A pathologist ranked the severity of root resorption by considering both the number and area of root resorption lacunae. There was almost no root resorption in the NC samples. Active orthodontic force induced moderate root resorption in the PC group. The ZA specimens had only a few resorption lacunae.

Number and area of root resorption lacunae significant decreased from PC to Z groups (P=0.02).

## Discussion

Inhibition of osteoclastic function is the main characteristic of the bisphosphonates family.^6-10^ Bone remodeling includes osteoblastic bone formation and osteoclastic bone resorption. Therefore, bisphosphonates can suppress the resorptive capacity of remodeling and preserve the bone mass in metabolic bone disease or bone malignancies. ZA is the most potent member of this group and its ideal efficacy has been previously documented for this purpose.^8^

### OTM

Despite the aforementioned advantages of ZA, a potent member of bisphosphonates, it did not have a significant effect on OTM in the present study. This result is in contrast to those of previous studies, which evaluated the effects of bisphosphonates on OTM and relapse prevention.^10-15^ Ortegaand colleagues^16^ designed a study similar to ours. They injected a single dose (16 μg) of ZA into the maxillary buccal vestibule next to the second molar tooth. They evaluated anchorage preservation by injection of ZA during space closure of first molar extraction site. They found that local application of a small single dose of ZA can maximize anchorage preservation and significantly prevent bone loss and root resorption.

We can rationalize this result by reviewing biodistribution of ZA in rats. Weiss et al.^18^ did a valuable experimental study on ZA biodistribution in rats and dogs. They evaluated systemic distribution of a single dose of 0.15 mg/kg ZA (intravenous injection) in rats weighing 190-250 g, and concluded that this dose is similar to the maximum dose tested in the oncology phase 3 clinical trials of 8 mg IV per patient (0.13 mg/kg for a 60-kg patient). Plasma concentration of the drug decreased rapidly after injection, and it was distributed in high perfusion organs. Bone absorbed the drug in the first day but drug concentration decreased in non-calcified tissues. The highest uptake was observed in cancellous bone and axial skeleton. The maxilla and mandible exhibited the same drug concentration as other non-calcified organs. Because of moderate binding to rat’s plasma proteins and hydrophobic characteristic of ZA, 36% of administered drug was excreted during the first 96 hours via urine.

Injection of 0.02 mg ZA per rat in the buccal vestibular mucosa was done locally during our study, based on the above-mentioned excretion percentage; thus, it can be expected that there was just 0.01 mg available drug at the end of day four. The four days after orthodontic force application are critical for OTM. Keles^15^ explained that progressive OTM occurs following osteoclastic recruitment. This takes place in day four after force application. Osteoclasts invade the pressure side and resorb alveolar bone in the force direction. As mentioned earlier, it seems that ZA concentration decreases by half because of moderate drug affinity to rat plasma proteins and probable rapid wash out from the soft tissue mucosa. Also, low uptake of drug by the maxillary bone was expected; therefore, we may conclude that adequate concentration of drug was not available in the right time to affect OTM.

Green^19^ demonstrated that ZA had the highest potency among bisphosphonates to inhibit bone resorption. IC_50_ reported 2 nM (0.002 μM).^20^ Kimachi^21^ in an in vitro study demonstrated that ZA inhibited TNF-α and RANKL-induced upregulation of RANK in osteoclast precursors in a dose-dependent manner. This may suppress bone resorption. They used 0.5 μM (544 μg) ZA as minimum dose, which can inhibit osteoclastogenesis. Other studies^22,23^ used higher doses for this purpose. The molecular weight of ZA is 272.09 g/mol (Available at: http://pubchem.ncbi.nlm.nih.gov/compound/Zoledronic_acid).^24^ As mentioned earlier, there was 0.01 mg (10 μg) expected drug dose in the fourth day, which is much lower than the lowest dose used by Kimachi et al (0.018 times).^21^ Ortega^16^ used 16 μg ZA in his study, which was 1.25 times lower than the dosage used in our study (20 μg)‏.ZA suppresses osteoclast precursors in a dose-dependent manner. Thus, we suppose that the efficacy of Zolena should be compared with the most effective ZA (Ortega's study with the lowest recommended dose) which was used in all studies in this field. Thus, we selected 20 µg dose in our study.

The controversy between the results of our study and that of Ortega may be due to the fact that ZA produced in Iran (Zolena) may have lower efficacy than the Zolendronate brand. Potency of a drug is a measure of the drug to produce a given response. Drug potency depends on both its affinity and efficacy. Affinity is the ability of the drug to bind to its receptors. Efficacy is the ability of the drug to initiate response after binding to its receptors. Although drug distribution was similar in our study and that of Ortega, the drug's affinity or efficacy might have been different in the two studies. The bisphosphonates’ potency is majorly affected by small changes in their structure, which can influence their ability to inhibit farnesyl diphosphate synthase in osteoclasts.^25,26^

We should consider that the mesial movement of the first molar may lead to simultaneous mesial movement of the second molar because of the forces applied to trans-septal fibers. This was a limitation of our study, which might have influenced the reliability of tooth movement measurement. It is suggested to use more accurate tools such as cone beam computed tomography (CBCT) or cephalometric measurements for this purpose.

Kirschneck et al^27^ explained that two-dimensional radiographs such as cephalograms have disadvantages such as object superimposition or low reproducibility (for proper identification of landmarks). Thus, cephalometric analysis was not performed in our study. However, Ortega (the most similar study to ours) used a leaf gauge and cephalometric measurement to analyze OTM; but both of these methods have disadvantages for this purpose.^16^

Kirschneck suggested CCD microscope camera and CBCT as reliable tools for analyzing OTM.^27^ Sirisoontorn et al^28^ used CBCT superimposition with special software (Ratoc System) for this purpose. We searched for similar software programs with the same ability, but there was no similar software in the radiology departments of dental schools in Iran. Lack of CBCT superimposition software was another limitation of our study.

Mirhashemi et al^29^ published a similar article about the effect of drug administration on OTM. They used a gauge for tooth movement measurement due to similar limitations. Finally, we decided to use a gauge for tooth movement assessment as the most commonly used tool for this purpose in similar studies.

#### Histological analysis

#### Howship’s lacunae

In the ZA group, mild distribution of resorptive lacunae was observed, and it was demonstrated that ZA significantly decreased the resorption process compared to PC group. This result suggested that although Zolena could not inhibit OTM significantly but had positive effects on bone preservation in histological level.

#### Blood vessels

ZA decreased vessel distribution to normal, which confirmed anti-angiogenic effect of bisphosphonates.^8^ Despite the possibility of occurrence of bisphosphonate-related osteonecrosis of the jaw,^9^ local administration of low doses of Zolena does not affect blood supply and oxygen delivery to maxillofacial bones. Therefore, this method of drug application canbe suggested with safety in order for orthodontic treatment control.

#### Osteoclast-like cells

The best way to evaluate osteoclast cells is to use tartrate-resistant acid phosphatase

(TRAP) staining.^30^ We had to use traditional morphometric evaluation of osteoclasts because of limited availability and higher cost of this specific staining.^11,31^ They can be distinguished as multinucleated cells on bone surfaces. Therefore, our results in this respect should be interpreted with caution.

The ZA injection was concomitant with significant decrease in osteoclast number; also, it was associated with a significant decrease in formation of Howship's lacunae. This finding was confirmed by previous studies,^10,11,32^ while it was in contrast to the results of some other studies,^14,33-37^ which indicated an increase in number of osteoclasts with reduced bone resorptive capability as the result of administration of bisphosphonates. Since histological staining was not specific for osteoclasts (TRAP), we cannot conclude that ZA decreases osteoclast population.

#### Root resorption

Bisphosphonate prescription led to a significant decrease in number and extent of root resorption lacunae. Although the PC group had moderate root resorption, group Z had mild resorptionand there was a significant difference between these groups (P<0.05). Thus, it can be concluded that ZA can significantly inhibit root resorption compared to the PC group in terms of extent and severity. This result suggested positive histological effect of ZA on inhibition of root resorption during OTM.^11^

## Conclusion

ZA had no significant inhibitory effect on OTM, while it significantly decreased bone and/or root resorption and angiogenesis compared to the PC group. It seems that ZA produced in Iran (Zolena) has less potency than its foreign made counterpart. The affinity and efficacy of Zolena should be investigated in future studies to find the minimum effective dose of this drug.

## Acknowledgments

We appreciate Dr. Negin Matini in order to help us in experimental procedure.

## Authors’ contributions

Massoud Seifi: Study design, supervision, data analysis.

Sohrab Asefi: Study design, experiment, data collection, data analysis, manuscript writing.

Ghazal Hatami Fard: Experiment, data analysis, manuscript writing.

Ali Lotfi: Experiment.

## Funding

Study was funded by Research Center, Research Institute of Dental Sciences, Shahid Beheshti University of Medical Sciences.

## Competing interests

The authors declare that they have no competing interests with regards to authorship or publication of this paper.

## Ethical approval

All of the experimental procedures were performed according to the approved protocol of the Institutional Animal Care and Usage Committee (ARRIVE guideline) and confirmed by the Ethical Committee of the Shahid Beheshti University of Medical Sciences.
